# RASSF1 Polymorphisms in Cancer

**DOI:** 10.1155/2012/365213

**Published:** 2012-05-31

**Authors:** Marilyn Gordon, Mohamed El-Kalla, Shairaz Baksh

**Affiliations:** ^1^Department of Pediatrics, Faculty of Medicine and Dentistry, University of Alberta, 3-055 Katz Group Centre for Pharmacy and Health Research, 113 Street 87 Avenue, Edmonton, AB, Canada T6G 2E1; ^2^Women and Children's Health Research Institute, University of Alberta, 4-081 Edmonton Clinic Health Academy, 11405-87 Avenue, Edmonton, AB, Canada T6G 1C9

## Abstract

Ras association domain family 1A (RASSF1A) is one of the most epigenetically silenced elements in human cancers. Localized on chromosome 3, it has been demonstrated to be a bone fide tumor suppressor influencing cell cycle events, microtubule stability, apoptosis, and autophagy. Although it is epigenetically silenced by promoter-specific methylation in cancers, several somatic nucleotide changes (polymorphisms) have been identified in RASSF1A in tissues from cancer patients. We speculate that both nucleotide changes and epigenetic silencing result in loss of the RASSF1A tumor suppressor function and the appearance of enhanced growth. This paper will summarize what is known about the origin of these polymorphisms and how they have helped us understand the biological role of RASSF1A.

## 1. Introduction

Cancer is a disease affecting 1 in 3 adults worldwide and is considered to be the second leading cause of death in both Canada and the United States behind heart disease [1, 2]. It is thought that cancer arises due to the occurrence of 2–5 genetic events to potentiate tumor formation and sustain abnormal growth [3]. These genetic changes occur in passenger genes (to support the cancer phenotype) and driver genes (to promote the cancer phenotype) [4]. About 10% of driver genes code for oncogenes that promote accelerated growth. However, about 90% of the driver genes code for tumor suppressor genes that inhibit accelerated growth [3], suggesting that tumor suppressor genes play an integral part in the origin of cancer. Evidence also suggests that the mutation rate of tumor suppressor genes are much higher than oncogenes supporting their importance in cancer formation [3, 5].

In 2000, Hanahan and Weinberg systematically described several key features or “hallmarks” of cancer that defined the behavior of a cancer cell [6]. These defining features of a cancer cell included the unique properties of limitless replicative potential, evasion of apoptosis, ability to stimulate neo-vascularization, invasion and metastasis, inhibition of suppressor pathways, and sustained proliferation. As described in their seminal paper, the aforementioned hallmarks are acquired through a “multistep process” that allows the cancer cells to acquire key survival traits while avoiding the watchful eye of established molecular “checkpoints” to inhibit abnormal growth [7]. It was around this time that the RASSF1 was identified as a potential tumor suppressor gene on chromosome 3, at 3p21.23 [8, 9]. Now more than a decade later, RASSF1A has been demonstrated using numerous approaches to be a tumor suppressor gene and an important driver gene in cancer influencing/intersecting with many of the hallmarks of cancer [8, 10]. It is epigenetically silenced in the majority of cancers by promoter specific methylation, resulting in loss of expression of the RASSF1A protein [11]. Although expression loss of RASSF1A by methylation occurs frequently in cancer, nucleotide changes by somatic mechanisms have also been detected in patients from several cancer subtypes. Several studies have tried to elucidate the importance of these polymorphic changes and how it may affect the tumor suppressor function of RASSF1A. They have also revealed interesting and surprising influences on numerous aspects of biology.

## 2. The Origin of RASSF1A Polymorphisms

**Figure 1:**
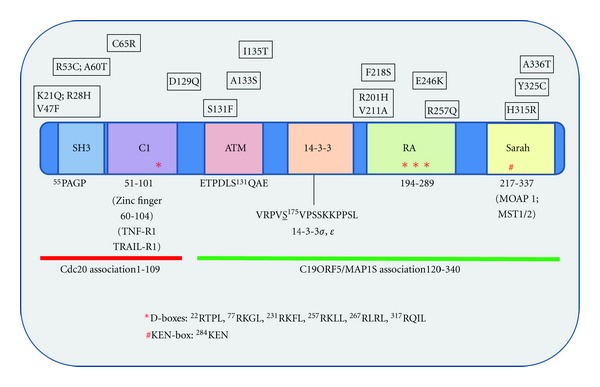
Schematic of RASSF1A with location of identified polymorphisms. Location of identified RASSF1A polymorphisms is indicated with respect to amino acid location, changed amino acid, and exon location. A potential binding sequence to an SH3 domain has been identified with a PxxP motif. The ATM phosphorylation site is underlined with surrounding residues shown. The docking sites for several RASSF1A effector proteins are shown including the location of potential D- and KEN-boxes for protein association (D1 to D6). The latter boxes are thought to be important for associations with APC/cdc-20 [12]. The Ras association domain (RA) is present in RASSF1A but has not been convincingly demonstrated to associate with the Ras family of oncogenes [10]. The SARAH domain modulates heterotypic associations with the sterile-20-like kinases, MST1 and MST2 (adapted from El-Kalla et al. (2010)) [13] and Gordon and Baksh (2011) [10].

The RASSF1 gene consists of eight exons alternatively spliced to produce 8 isoforms, RASSF1A-H, that have distinct functional domains including the Ras association (RA) domain [9, 14]. Of these, RASSF1A and RASSF1C are the predominant ubiquitously expressed forms in normal tissues [9, 11]. RASSF1C has been demonstrated to be perinuclear in appearance in NCI H1299 lung cancer cells [15], nuclear in HeLa cells with translocation to the cytosol upon DNA damage [16], and localized to microtubules in a similar fashion to RASSF1A in 293T cells [17, 18]. Thus, the localization of RASSF1C is varied and controversial. This is not the case for RASSF1A as it has been demonstrated by our group and several others to be a microtubule binding protein having a microtubule like localization and functioning to stabilize tubulin in a taxol like manner [13, 18, 19]. To date, a crystal structure for RASSF1A or RASSF1C has not been identified, but Foley et al. [20] provided a molecular model of the N-terminal C1 domain containing four zinc finger motifs which is very similar to the one found on RASSF5A/Nore1A [21]. The zinc finger motifs have now been demonstrated to be involved in death receptor associations and possible associations with other receptors or signaling components [20]. In addition to the C1 domain, RASSF1A has been noted to have a sequence specificity motifs to associate with SH3 domain (PxxP); motifs for 14-3-3 associations; a Ras association (RA) domain (although association is weak or indirect for K-Ras) [10]; associations with the anaphase promoting complex protein cdc20 and the autophagy protein C19ORF5/MAP1S; and heterotrophic associations with the Hippo proapoptotic kinase (MST1/2) and the BH3-like protein modulator of apoptosis 1 (MOAP-1) through the Salvador/RASSF/Hippo (SARAH) domain (both reviewed elsewhere in this issue) (please see Figure 1 for schematic summary of RASSF1A protein associations).

RASSF1A polymorphisms have been identified in several cancers as listed in Table 1 and can be mapped to specific protein interaction domains (Figure 1). These polymorphisms have been found in tumors from numerous cancer patients and cell lines [22]. The population distribution and significance of these alterations in tumorigenesis remain to be determined but do vary from 9% to 33% of the specific cancer population. The majority of RASSF1A polymorphisms have been confirmed using several approaches as outlined by the 1000Genome project (http://www.1000genomes.org/), HapMap project (http://hapmap.ncbi.nlm.nih.gov/) and submitted by multiple sources (Table 1 and NCBI SNP database [http://www.ncbi.nlm.nih.gov/projects/SNP/snp_ref.cgi?showRare=on&chooseRs=coding&go=Go&locusId=11186] and University of Maryland SNP database [http://bioinf.umbc.edu/dmdm/gene_prot_page.php?search_type=gene&id=11186,NP_009113]). Recently, a comprehensive study of 400 lung, renal, breast, cervical, and ovarian cancers by Kashuba et al. [22] revealed frequent loss of genetic material on chromosome 3p in 90% of the tumors investigated. Furthermore, they determined that the mutation rate in cancer for RASSF1A was 0.42 mutation frequency/100 base pair whereas in the “normal” population was about 0.10 mutations/100 base pairs. They speculate that RASSF1A has a 73% GC content within exons 1–2 which may explain the high mutation rate of RASSF1A within cancer cells. Within cell lines, RASSF1A was found to carry 0.7 mutations/100 bp in the Burkitt's lymphoma-derived cell lines, BL2 and RAMOS, whereas it was 0.14 in the renal carcinoma cell line KRC/Y and, with each division of the BL2 lymphoma line, transitional mutations were observed. Interestingly, codon changes in RASSF1A were also observed in 15 normal human hearts that included two nucleotide changes (CTA to CTG and GTA to GTG) but no amino acid changes [22]. They speculate that RASSF is simply located in an area that is “extensively damaged” and susceptible to mutational pressures in 90% of epithelial cancers [22].

The most common polymorphism is the alanine (A) to serine (S) at amino acid 133 (A133S) located within the ATM DNA damage checkpoint kinase site (please see below sections). This has been identified as a single nucleotide germ line polymorphism (SNP) on both alleles in some breast cancer patients and is significantly associated with BRAC1/2 mutations. Patients with wild-type BRAC1/2 and RASSF1A A133S have a +15-year better survival period than those harboring both BRAC1/2 mutations and RASSF1A A133S [23, 29]. The RASSF1A A133S SNP has been found in 20.6% of patients with breast carcinomas [23, 29], 19.8% in lung cancer [29, 32], 11.1% in head and neck cancer [32], 6.9% in colorectal cancer [32], 14.3% in esophageal cancer [32], 24.3% in patients with fibroadenoma and in 2.9%–10% of healthy controls [23, 32]. Interestingly, Gao et al. [29] also revealed the presence of the A133S polymorphism in brain and kidney cancer patients and Bergqvist et al. (2010) detected the presence of the A133S SNP in 18.4% of the white British female population [28]. The high percent obtained for the latter is surprising and requires further validation. The prevalence of the rest of the RASSF1A polymorphisms has not been determined yet, and functional studies to systematically determine influence of these polymorphisms on RASSF1A biological function are yet to be done. However, in this paper we will only summarize what has been carried out already to ascertain the consequences of polymorphisms to RASSF1.

## 3. RASSF1A: A Key Element in Cellular Stability

**Figure 2:**
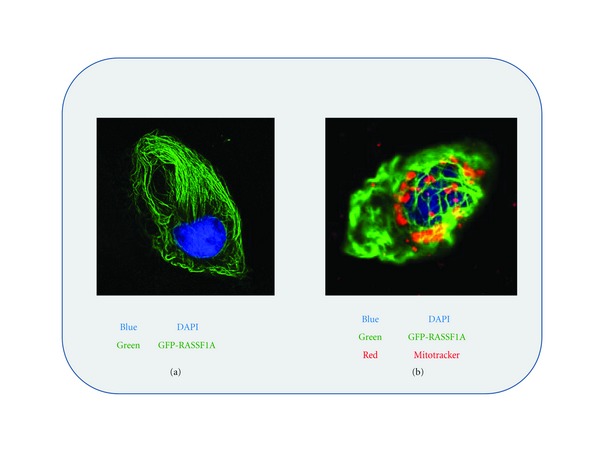
Microtubule localization of RASSF1A. GFP-RASSF1A was expressed in U2OS osteosarcoma cells (a and b) and costained with DAPI to reveal the nucleus (a and b) and with mitotracker red to reveal mitochondrial localization (b). Areas of yellow reveal colocalization and all images were acquired using confocal microscopy using a Zeiss system and a 63x oil immersion lens.

One of the most striking features of RASSF1A is its microtubule appearance. Numerous tagged versions of RASSF1A have all revealed similar microtubule-like appearance as seen in MCF-7 breast cancer cells in Figure 2. This appearance has been observed in many other cell lines with similar appearances. It has also been determined that both N- and C-terminal residues of RASSF1A are required for the microtubule appearance of RASSF1A [13, 18]. Several groups have characterized the appearance and function of the microtubule localization of RASSF1A. It has been demonstrated that the microtubule localization of RASSF1A mainly functions to stabilize tubulin both in interphase and in mitosis even in the presence of the microtubule destabilizer, nocodazole [13, 27, 33, 34]. To date, RASSF1A has not been demonstrated to colocalize to actin or intermediate filaments. RASSF1A associations function to stabilize tubulin in a paclitaxel (taxol)-like manner [17, 27] especially during mitosis allowing sister chromatid segregation. This function is governed by associations with *γ*-tubulin at spindle poles and centromeric areas during metaphase and anaphase and near the microtubule organizing center (MTOC) where microtubules emerge and nucleate [35–37]. If the microtubule spindle complex is not properly formed, cell death proceeds to prevent inheritable aneuploidy. In the absence of cell death pathways chromosomal missegregation and inheritable aneuploidy arise which can lead to malignancy. Several of the effects on microtubule biology have been observed in mouse embryonic fibroblasts (MEFs) obtained from *Rassf1a^−/−^* mice developed by two separate groups [19, 38]. *Rassf1a^−/−^* mice are viable, fertile and retain expression of isoform 1C. However, by 12–16 months of age they have increased tumor incidence, especially in the breast, lung, and immune system (gastrointestinal carcinomas and B-cell-related lymphomas) [19, 38]. These data suggest a tumor suppressor function specific for the RASSF1A isoform. MEFs obtained from *Rassf1a^−/−^* mice are more susceptible to nocodazole-induced microtubule depolymerization suggesting a protective effect of RASSF1A on microtubule stability similar to what has been observed using tissue culture approaches [19].

 It has now been demonstrated that RASSF1A disease associated polymorphisms may affect the function of RASSF1A as a microtubule stabilizer. It was demonstrated that the S131F mutant of RASSF1A continued to maintain the ability to promote tubulin stability as determined by immunofluorescence microscopy and acetylation status of tubulin [17]. Furthermore, it was demonstrated by Vos et al. [17] that RASSF1C could not function in a similar manner to RASSF1A to stabilize tubulin. This provided one of the first evidences for differential function for these two prominent isoforms of the RASSF1 loci. A comprehensive analysis of several other RASSF1A polymorphisms was carried out by Liu et al. [39]. They demonstrated that polymorphisms around the ATM phosphorylation site (A133S, S131F, and I135T) maintained the microtubule appearance of RASSF1A. A second comprehensive study revealed that the C65R and R257Q polymorphisms of RASSF1A resulted in “atypical localizations” of RASSF1A away from a microtubular appearance [27]. Furthermore, both C65R (a residue within the C1 domain) and R257Q (a residue within the RA domain) promoted enhanced BrdU incorporation into NCI-H1299 nonsmall cell lung cancer cells suggesting loss of tumor suppressor function. Recently, we have also observed a complete loss of the microtubule localization of RASSF1A in the presence of a C65R change and “oncogenic” properties of this polymorphism in a classical xenograft assay in athymic mice [13]. The C65R polymorphism acquired a nuclear localization for unexplained reasons and also failed to stabilize tubulin in the presence of the microtubule depolymerizing agent nocodazole [13]. It clearly lost the tumor suppressor function of RASSF1A in a xenograft assay and can robustly drive enhanced growth [13]. Similarly, both the A133S and E246K mutants maintained microtubule localization and lost tumor suppressor function but not to the level of the C65R polymorphism (xenograft assays were carried out in HCT116 colon cancer cells) [13]. We are currently characterizing many of the other polymorphisms in Table 1 for their ability to behave as tumor suppressor, inhibit abnormal growth, and affect microtubule stability and protein interaction with established RASSF1A effectors.

Interestingly, it has been reported that Epstein-Barr virally encoded protein, latent membrane protein 1 (LMP1) can function to transcriptionally decrease RASSF1A levels and promote tubulin depolymerization and mitotic instability in human epithelial cells (HeLa and HaCaT) [40]. Punctuate structures of tubulin were observed in the cytoplasm indicative of tubulin depolymerization [40]. Decreased RASSF1A levels resulted in increased phosphorylation of I*κ*B*α* and elevated NF*κ*B activity. Cause and effect of changes in NF*κ*B activity were not fully elucidated. However, we have evidence that the loss of RASSF1A can lead to enhanced NF**κ**B activity (El-Kalla et al., unpublished observations) suggesting that the decreased expression of RASSF1A induced by LMP1 production may have resulted from the loss of the ability of RASSF1A to restrict NF**κ**B function. EPV infection is closely related to the appearance of nasopharyngeal cancers and we speculate that a precondition characterized by enhanced NF*κ*B activity (and hence inflammation) may promote tumorigenesis and the appearance of nasopharyngeal cancers upon EBV infection. We are currently exploring the role of RASSF1A as a molecular link between inflammation and tumorigenesis.

## 4. RASSF1A: Linking Extrinsic Death Receptor Stimulation to Bax Activation

Every cell has an inherent ability to die under abnormal conditions. This ability has been programmed by nature into every cell and follows a defined series of events. Apoptosis is critical for multiple physiological processes, including organ formation, immune cell selection, and inhibition of tumor formation [41]. Two types of signaling pathways promote apoptosis using the mitochondria. The “intrinsic” pathway is activated by noxious factors such as DNA damage, unbalanced proliferative stimuli, and nutrient or energy depletion. Components of intrinsic-dependent apoptosis are still unclear, although Bcl-2-homlogy domain 3 (BH3) proteins are required. In contrast, the “extrinsic” pathway is stimulated by specific death receptors (e.g., TNF*α* receptor R1 (TNF-R1), TNF*α*-related apoptosis-inducing ligand receptor (TRAIL-R1) or Fas (CD95)) [42–44]. Molecular mechanisms modulating programmed cell death (apoptosis) impinge on growth and immune cell function. We speculate that these cellular processes may be regulated in part by tumor suppressor pathways, pathways frequently inactivated in several disease states (such as cancer and autoimmune/inflammatory disorders).

RASSF1A is one element involved in death receptor-dependent cell death that is epigenetic-silenced in numerous cancers. In the majority of these studies, RASSF1A epigenetic silencing strongly correlates with the epigenetic silencing of three other genes—p16^INK4a^, death associated protein kinase (DAPK), and caspase 8 [45–48]. Two of these genes are involved in proapoptotic pathways, DAPK and caspase-8 [43, 49, 50]. DAPK is a unique calcium/calmodulin activated serine/threonine kinase involved in several cell death-related signaling pathways including tumor necrosis factor *α* receptor 1 (TNF-R1) cell death and autophagy [50, 51]. It is a tumor suppressor protein [50] that has also been demonstrated to be involved in associations with and the regulation of pyruvate kinase, a key glycolytic enzyme that may be influential in the link between metabolism and cancer [52]. We have evidence to demonstrate association of RASSF1A and DAPK (Baksh et al., unpublished observations) and RASSF1A has two potential phosphorylation sites for DAPK within the RA domain at ^193^GRGTSVRRRTSFYLPK [53]. Curiously, these sites have also been demonstrated to be sites for protein kinase C [54] and aurora kinases [55]. In the presence of S197A or S203A mutant of RASSF1A, PKC failed to phosphorylate RASSF1A resulting in the loss of microtubule organization in COS-7 cells. Similarly, Aurora B kinase failed to phosphorylate RASSF1A in the presence of S203A resulting in a failed cytokinesis [55]. It remains to be determined the physiological importance of these potential DAPK phosphorylation sites.

Caspase 8 is cysteine-dependent aspartate-directed protease and an initiator caspase, and targeted activation of caspase 8 is driven by the disc inducing signaling complex (DISC) [43, 49]. DISC-dependent activation of caspase 8 triggers a series of events resulting in the cleavage of Bid and insertion of Bid on the outer mitochondrial membrane, the release of small molecules (such as cytochrome c) from the mitochondria into the cytosol, and the activation of downstream effector caspases (such as caspase-3) [56]. Intrinsic pathway stimulation also leads to cytochrome c release and effector caspase activation. Once activated, effector caspases cleave several proteins (such as poly(ADP-ribose) polymerase (PARP)) and activate specific DNA endonucleases resulting in nuclear and cytoplasmic breakdown [57].

**Figure 3:**
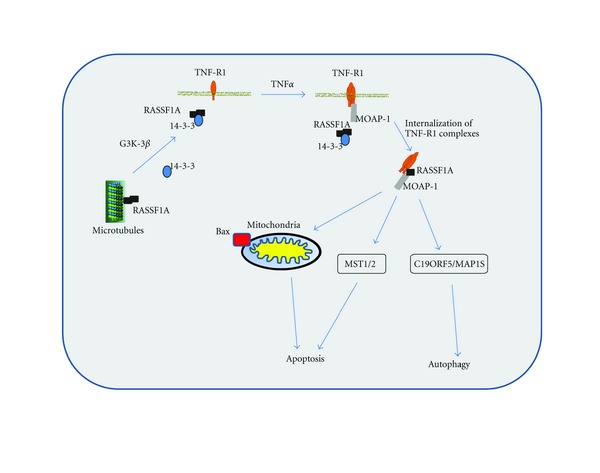
Model for the RASSF1A/MOAP-1 proapoptotic pathway. Death receptor-induced cell death (TNF*α* is used as an example) can result in the recruitment of protein complexes to activate Bax and promote apoptosis. Basally, RASSF1A is kept complexed with 14-3-3 by GSK-3*β* phosphorylation in order to prevent unwanted recruitment of RASSF1A to death receptor and uncontrolled stimulation of Bax and apoptosis. Once a death receptor stimuli have been received (TNF*α* as shown above), the TNF-R1/MOAP-1/RASSF1A complex promotes the “open” form of MOAP-1 to associate with Bax. This in turn results in Bax conformational change and recruitment to the mitochondria to initiate cell death. Following release from TNF-R1/MOAP-1 complex, RASSF1A may reassociate with 14-3-3 to prevent continued stimulation of this cell death pathway (unpublished observations). Please see text for further details.

Our research group was the first to define and continues to define some of the molecular mechanisms of death receptor-dependent apoptotic regulation by RASSF1A [20, 58, 59]. Ectopic expression of RASSF1A (but not RASSF1C) specifically enhanced death receptor-evoked apoptosis stimulated by TNF*α* that does not require caspase 8 activity or Bid cleavage [20, 58]. We have also shown that RASSF1A does not influence the intrinsic pathway of cell death [58]. Furthermore, we demonstrated that microtubule localization was required for association with death receptors and for the role of RASSF1A in apoptosis [13, 20]. In contrast, RASSF1A knockdown cells (by RNA interference) and *Rassf1a^−/−^* knockout mouse embryonic fibroblasts (MEFs) have significantly reduced caspase activity, defective cytochrome c release and Bax translocation (but not Bid cleavage), and impaired death receptor-dependent apoptosis [58]. These data suggest a direct link of death receptor activation of Bax through RASSF1A. Our current model of RASSF1A-mediated cell death is described in Figure 3. Death receptor stimulation functions to bring RASSF1A (and not RASSF1C or RASSF5A/Nore1A) and modulator of apoptosis 1 (MOAP-1) to TNF-R1 in order to promote a more “open” MOAP-1 to subsequently associate and promote Bax conformational change and translocation to the mitochondria to activate cell death (Figure 3) [20, 58–60]. We have evidence that the 14-3-3 may keep RASSF1A in check and inhibit it from promoting cell death or associating with other unexplored signaling components [59]. We are currently characterizing the primary and secondary signals required for MOAP-1 induced Bax conformational change and the apoptotic regulation of MOAP-1 by ubiquitination (Law et al., unpublished observations).

To date, very little is known about the cell death properties of numerous RASSF1A polymorphisms. Dallol et al. demonstrated that both C65R and R257Q promoted enhanced BrdU incorporation into NCI-H1299 non-small cell lung cancer cells suggesting loss of tumor suppressor function and possible loss of cell death properties [27]. We have observed partial activation of apoptosis in the presence of several RASSF1A polymorphisms (such as C65R, A133S, I135T, and A336T) suggesting importance to death receptor-dependent apoptosis via ATM site and SARAH domain associations (El-Kalla et al., unpublished observations). Further analysis is warranted to explore how RASSF1A polymorphisms may affect death receptor-dependent apoptosis.

Although not discussed in great detail here, RASSF1A can also promote cell death utilizing the autophagic protein, C19ORF5/MAP1S, [27, 33, 61] the Hippo pathway components MST1/2 and possibly Salvador [14, 62], and, in melanoma cells, influence Bcl-2 levels and activate apoptosis signal regulating kinase 1 (ASK-1) [63]. Min et al. [33] demonstrated that the ability of RASSF1A to efficiently inhibit APC/cdc20 activity during mitosis (please see next section) is dependent on the recruitment of RASSF1A to spindle poles via C19ORF5/MAP1S. C19ORF5/MAP1S was also shown to regulate mitotic progression by stabilizing mitotic cyclins in a RASSF1A-dependent manner. Recently, C19ORF5/MAP1S was demonstrated by Lui et al. [61] to associate with a component of the autophagosome, LC3, and the mitochondria-associated leucine-rich PPR-motif containing protein (LRPPRC) protein. These associations suggest that C19ORF5/MAP1S may serve as a potential link between autophagic cell death, mitochondria, and microtubules and appears to require RASSF1A. It will be essential to determine associations of RASSF1A polymorphisms with key cell death mediators, such as MOAP-1, TNF-R1, DAPK, C19ORF5/MAP1S, and MST1/2 in order to ascertain their importance in influencing the tumor suppressor function of RASSF1A. A detailed discussion about the Hippo and RASSF1A/MOAP-1 pathways of cell death is presented in this special review.

## 5. Cell Cycle Control Pathways Influenced by RASSF1A

As mentioned previously, RASSF1A is a microtubule binding protein that colocalizes with *α*- and *β*-tubulin, and with *γ*-tubulin on centromeres [35–37]. RASSF1A is thought be an important component of mitotic spindles and can influence the separation of sister chromatids at the metaphase plate. This observation has held true five years later and reinforced the findings of Song et al. [64] of the possible involvement of RASSF1A in cell cycle control. Although, very limited knowledge of the cell cycle effects of polymorphic forms of RASSF1A are known, several lines of evidence do suggest a role in cell cycle control. In 2004, RASSF1A was identified as an interacting protein with the anaphase promoting complex (APC)/cdc20 and prevented the ability of APC/cdc20 to degrade cyclins A and B in order to exit mitosis [64]. In the absence of RASSF1A, cyclins A and B were rapidly degraded due to increased ubiquitination of the cyclins to allow exit from mitosis.

Whitehurst et al. [65] supported this role for RASSF1A and further identified *β*-TrCP as associating with RASSF1A and functioning to restrict the role of APC-cdc20 in mitotic progression. *β*-TrCP is an I*κ*B*α* E3 ligase and negative regulator of the *β*-catenin/WNT signaling pathway. Although Liu et al. could not find evidence for a RASSF1A-APC/cdc20 association [66], the influence of RASSF1A on APC/cdc20 was once again demonstrated by Chow et al. in 2011 [67]. They not only demonstrated an association with APC/cdc20, but also clearly showed that a “RASSF1A-APC/cdc20 circuitry” was in place in HeLa cells to regulate mitosis. RASSF1A associates with APC/cdc20 via two D boxes at the N-termini (DB1 and DB2) and keeps it inhibited until there is mitotic activation of the serine/threonine kinases Aurora A/B. Phosphorylation of RASSF1A by Aurora A/B on T202 or S203 subsequently labels RASSF1A as a target to the E3-ubiquitin ligase activity of APC, ensuring that mitosis proceeds by degrading RASSF1A and suppressing its mitotic inhibitor function [12]. They speculate that this occurs before spindle body formation and sister chromatid separation. Their results are intriguing and reveal the complex signaling world that RASSF1A is part of.

Beyond a RASSF1A-APC/cdc20 molecular control of mitosis, research has continued into a potential role of RASSF1A during cell cycle progression. This has led to several observations suggesting RASSF1A G1/S regulation of cyclin D1 [63, 65, 68] in melanoma and HeLa cells (resp.), interaction with the transcriptional regulator p120^E4F^ at the G1/S phase transition resulting in inhibition of passage from G1 [69], DNA damage control regulation by ATM and by the DNA damage binding protein 1 (DDB1) that can associate with RASSF1A linking to the E3-ligase cullin 4A during mitosis [70]. The p120^E4F^ transcription factor was determined to be involved in inhibiting the transcription of cyclin A, resulting in the failure of cyclin A to associate with CDK2 to allow for progression through S phase. RASSF1A cooperates with p120^E4F^ to repress cyclin A expression by enhancing its binding at the promoter region [69]. ATM and DDB1 are important DNA damage control elements during ultraviolet and gamma irradiation which have evolved to repair damage DNA and will be discussed elsewhere in this special RASSF issue.

**Figure 4:**
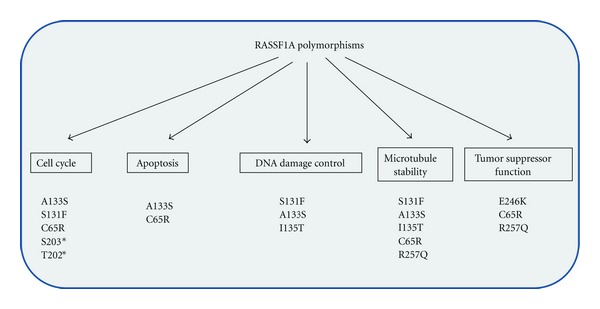
Identified biological roles for RASSF1A polymorphisms. Several polymorphisms have been identified for RASSF1A over the past decade since it was first cloned. Biological analyses of the *in vivo* role have identified the importance of RASSF1A over numerous pathways. This figure summarizes what is known about RASSF1A polymorphisms. *denotes a nonpolymorphic but mutational change. This change does not naturally exist in the cancer patient population to our knowledge.

Shivakumar et al. revealed that in both H1299 non-small cell lung cancer and in the human mammary epithelial telomerase immortalized (HME50-hTERT) cell line, overexpression of RASSF1A wild-type expression construct can reduce BrDU accumulation and cyclin D1 expression [68]. The ability of RASSF1A to inhibit growth, and cyclin D1 expression was lost in the presence of the A133S and S131F ATM site mutants of RASSF1A suggesting an important role in tumor suppression [68]. Other polymorphic forms of RASSF1A have not been explored with respect to their abilities to regulate mitosis. What these studies reveal is how highly regulated RASSF1A is, not only in interphase cells, but especially in cells undergoing active cell division. It can then be appreciated how devastating the functional consequence of the loss of RASSF1A would be resulting in an unregulated and unwanted increase in mitotic cyclins, accelerated mitosis, enhanced growth and tumor formation. It would be interesting to speculate that they may result in the loss of the ability of RASSF1A to properly regulate mitosis and inhibit unwanted proliferation. It is imperative that we understand completely how polymorphic changes in RASSF1A may influence the important role of RASSF1A in mitosis and other biological pathways (Figure 4).

## 6. The DNA Damage Connection

One of the first motifs identified on RASSF1A was the phosphorylation site for the DNA damage serine/threonine kinase Ataxia telangiectasia mutated (ATM). ATM is usually activated and recruited in response to double strand breaks. It is part of a DNA damage checkpoint that ensures that damaged DNA is repaired in a timely and efficient manner. RASSF1A has been shown by several groups to be phosphorylated by ATM and the ATM site polymorphisms are present in several cancer types [71]. Although not currently well defined, RASSF1A is believed to have an important role in DNA damage control as evidenced by associations with xeroderma pigmentosum complementation group A (XPA) [72] and phosphoregulation by ATM [71, 73]. XPA is involved in nucleotide excision repair and association with RASSF1A has only been identified in a yeast two-hybrid screen [24]. Hamilton et al. [71] elucidated a novel pathway linking ATM-dependent phosphorylation of RASSF1A in response to gamma irradiation on serine-131 followed by MST/LATS activation resulting in Yes associated protein (YAP)/p73-dependent transcriptional program to promote cell death. The S131F mutant of RASSF1A lacked the ability to carry out the transactivation of YAP/p73. Curiously, RASSF1C has been demonstrated to be constitutively anchored to the death domain-associated protein (DAXX) in the nucleus and is released upon UV-induced DNA damage [16]. Localization with DAXX occurs on promyelocytic leukaemia-nuclear bodies (PML-NBs). DNA damage promotes the degradation and ubiquitination of DAXX, release of RASSF1C to allow the nucleocytoplasmic shuttling of RASSF1C to cytoplasmic microtubules, and the activation of the SAPK/JNK pathway in HeLa cells. RASSF1A was shown to only associate weakly with DAXX suggesting a specific role for RASSF1C [16]. Recently, it was demonstrated that the E3 ligase, Mule, can ubiquitinate RASSF1C under normal conditions, and both Mule and *β*-TrCP can ubiquitinate RASSF1C under UV exposure [74]. These studies and others have continued to demonstrate the diverse role that the splice variants of RASSF1 may function in biology. A detailed discussion about the role of RASSF1A during DNA damage repair will be presented in this special review.

## 7. RASSF1C: The Other RASSF1 Isoform

Very little is known about the biological role for the other major splice variant of the RASSF1 gene family. Several lines of evidence suggest that RASSF1C may be a tumor suppressor gene in prostate and renal carcinoma cells but not in lung cancer cells [75]. In fact, it has been demonstrated by Amaar et al. that the loss of RASSF1C actually results in the loss of proliferation of lung and breast cancer cells suggesting a prosurvival (not tumor suppressor) role for RASSF1C [76, 77]. Furthermore, RASSF1C can associate with the E3 ligase *β*-TrCP via the SS_18_GYXS_19_ motif (where X is any amino acid and numbers correspond to amino acid sequence in RASSF1C) at the N-terminus (i.e., not present in RASSF1A) [78] and promote the accumulation and transcriptional activation of *β*-catenin [78]. Activation of *β*-catenin would result in enhanced proliferation by transcriptional upregulation of genes such as cyclin D1, Myc, and TCF-1. Thus, either the lack of RASSF1A expression or the overexpression of RASSF1C perturbs *β*-TrCP E3 ligase/*β*-catenin homeostasis and WNT signaling pathways.

Unlike RASSF1A, RASSF1C has not been found to be significantly epigenetically silenced in cancer. Polymorphisms to RASSF1C have not been uncovered yet, but a C61F mutation in RASSF1C (equivalent to the S131F mutation in RASSF1A) resulted in the failure of RASSF1C to protect microtubules against nocodazole-induced depolymerization [17]. This would again suggest importance of serine residue within the ATM site found on both RASSF1A and 1C. Recently, it has been suggested that a possible pathogenic role for RASSF1C in cancer may exist as its expression was more than eleven-fold greater in pancreatic endocrine tumors than in normal tissue [79]. It remains to be determined the exact biological role for RASSF1C, but the ability of RASSF1C to function as a tumor suppressor is cell specific and remains to be further investigated and confirmed.

## 8. The Future of Understanding RASSF Polymorphisms

Knudson stated in 1971 that cancer is the result of accumulated mutations to the DNA of cells and that multiple “hits” to DNA were necessary to cause cancer [80]. It is generally known that the loss of function in a tumor suppressor protein typically requires the inactivation of both alleles of its gene in contrast to proto-oncogenes which promote tumorigenesis due to dominant acting mutations affecting one gene copy. Similar to what Knudson discovered for retinoblastoma, the RASSF1A tumor suppressor may become inactivated by the epigenetic loss by promoter specific methylation of both allele or by a combination of epigenetic silencing and loss of function polymorphic changes. Most cancers investigated to date have >50% of the disease population containing epigenetic silencing of RASSF1A [11, 81]. However, numerous cancers such as cervical, head and neck, myeloma, and leukemia have <25% of the disease population containing epigenetic silencing of RASSF1A. It may be speculated that polymorphic changes to RASSF1A may exist in the latter patients that, in agreement with the Knudson two hit hypothesis, resulting in the loss of function of the RASSF1A tumor suppressor and causing cancer. A systematic and functional analysis of RASSF1A polymorphism is therefore necessary to allow physicians to carry out personalized medicine on patients harboring polymorphic changes to RASSF1A.

## Figures and Tables

**Table 1:** RASSF1A single nucleotide polymorphisms. Several RASSF1A polymorphic changes have been identified as outlined in Table 1. SNP sites consulted to draft this table include NCBI (at http://www.ncbi.nlm.nih.gov/projects/SNP/snp_ref.cgi?showRare=on&chooseRs=coding&go=Go&locusId=11186) and DMDM (at http://bioinf.umbc.edu/dmdm/gene_prot_page.php?search_type=gene&id=11186).

Polymorphism	Tissue or cell line origin	SNP ID^%^ and other information	References
K21Q (AAG →CAG)	Breast (tumor) Kidney (renal carcinoma cell TK10 and KRC/Y) Lung (Non small cell Lung cancer cell line)	rs4688725^∗, ∗∗, #,^	Schagdarsurengin et al. [23]; Dammann et al. [24]; Agathanggelou et al. [25]; Burbee et al. [26]

R28H (CGT → CAT)	Breast (Tumor) Lung (nonsmall cell lung cancer cell line)	Presence in lung carcinomas are rare	Schagdarsurengin et al. [23]; Dammann et al. [24]; Burbee et al. [26]

V47F (GTC → TTC)	Not listed	rs61758759^∗, ∗∗, #,^	NCB1^%^

R53C (CGC →TGC)	Breast (tumor) Lung (nonsmall cell lung cancer cell line)	Q9NS23^$^	Schagdarsurengin et al. [23]; Dammann et al. [24]; Burbee et al. [26]

A60T (GCA → ACA)	Breast	No SNP ID found	Agathanggelou et al. [25]

C65R (TGC → CGT)	Breast (tumor)	No SNP ID found	Dallol et al. [27]

S131F (TCT → TTT)	Breast (tumor) Kidney (Wilm's tumor)	No SNP ID found	Schagdarsurengin et al. [23]; Dammann et al. [24]

A133S (GCT → TCT)	Breast (tumor, fibroademonas), 33% Kidney (Wilm's tumor), 21% Brain (medulloblastoma), 9% Muscle (rhadomyosarcoma), 19% Lung (nonsmall cell lung cancer cell line)	rs52807901 and rs2073498 Association with BRAC1/2 mutations Homozygous in breast cancer 21% of kids with germ line mutation, maternal in origin	Schagdarsurengin et al. [23]; Dammann et al. [24]; Bergqvist et al. [28]; Gao et al. [29]; Burbee et al. [26]; Lusher et al. [30]

I135T (ATT →ACT)	Lung (nonsmall cell lung cancer cell line) Breast (tumor cell line)	No SNP ID found	Dammann et al. [24]; Agathanggelou et al. [25]

V211A (GTC → GCC)	Breast	No SNP ID found	Agathanggelou et al. [25]

R201H (CGC → CAC)	ENT (nasopharyngeal carcinoma)	In 23 tumor samples, 34 other polymorphisms were detected (not listed in this table) with 30 transitions, 2 transversions, and 2 deletions (6 in SH3/C1 domain and 6 in RA domain)	Zhi-Gang Pan et al. [31]

E246K (GAA → AAG)	Breast (tumor)	No SNP ID found	Agathanggelou et al. [25]

R257Q (CGG → CAG)	Breast (Tumor) Lung (nonsmall cell lung cancer cell line	No SNP ID found	Schagdarsurengin et al [23]; Dammann et al. [24]; Agathanggelou et al. [25]; Dallol et al. [27]

H315R (CAC → CGC)	NCBI SNP database, source unknown	rs52792349 and rs12488879	Geoffery Clark (personnel communication)

Y325C (TAT → TGT)	Breast (tumor) Lung (nonsmall cell lung cancer cell line	No SNP ID found	Schagdarsurengin et al. [23]; Dammann et al. [24]; Burbee et al. [26]

L270V (CTG → GTG)	Cervical (tumor)	No SNP ID found	Schagdarsurengin et al. [23]; Dammann et al. [24]

A336T (GCC → ACC)	Lung (nonsmall cell lung cancer cell line	No SNP ID found	Dammann et al. [24]

^%^ http://bioinf.umbc.edu/dmdm/gene_prot_page.php?search_type=gene&id=11186.

^**$**^UniProtKB/Swiss-Prot.

*Validated by multiple, independent submissions to the refSNP cluster.

**Validated by frequency or genotype data: minor alleles observed in at least two chromosomes.

^#^Validated by the 1000 Genomes Project, http://www.1000genomes.org/.

## Abbreviations

APC: Anaphase promoting complex
ATM: Ataxia telangiectasia mutated
BH3: Bcl-2-homlogy domain 3 (BH3)
DAPK: Death-associated protein kinase
C19ORF5/MAP1S: Chromosome 19 open reading frame 5/microtubule-associated protein 1S
Nore: Novel ras effector
PKC: Protein kinase c
RAS: Rat Sarcoma
RASSF: Ras association domain family
RA: Ras association domain
NF*κ*B: Nuclear factor of kappa light polypeptide gene enhancer in B-cells
MAP: Mitogen activated kinase
MOAP-1: Modulator of apoptosis
SARAH: Salvador/RASSF/Hippo domain
TNF*α*: Tumor necrosis factor *α*
TNF-R1: TNF*α* receptor 1.

## References

[1] Canada S (2011). *Leading Causes of Death in Canada*.

[2] Minino AM (2009). Death in the United States.

[3] Bozic I, Antal T, Ohtsuki H (2010). Accumulation of driver and passenger mutations during tumor progression. *Proceedings of the National Academy of Sciences of the United States of America*.

[4] Vogelstein B, Kinzler KW (2004). Cancer genes and the pathways they control. *Nature Medicine*.

[5] Wang F, Tan W, Guo D, Zhu X, Qian K, He S (2010). Altered expression of signaling genes in jurkat cells upon FTY720 induced apoptosis. *International Journal of Molecular Sciences*.

[6] Hanahan D, Weinberg RA (2000). The hallmarks of cancer. *Cell*.

[7] Hanahan D, Weinberg RA (2011). Hallmarks of cancer: the next generation. *Cell*.

[8] Richter AM, Pfeifer GP, Dammann RH (2009). The RASSF proteins in cancer; from epigenetic silencing to functional characterization. *Biochimica et Biophysica Acta*.

[9] van der Weyden L, Adams DJ (2007). The Ras-association domain family (RASSF) members and their role in human tumourigenesis. *Biochimica et Biophysica Acta*.

[10] Gordon M, Baksh S (2011). RASSF1A: not a prototypical Ras effector. *Small Gtpases*.

[11] Donninger H, Vos MD, Clark GJ (2007). The RASSF1A tumor suppressor. *Journal of Cell Science*.

[12] Mochizuki T, Furuta S, Mitsushita J (2006). Inhibition of NADPH oxidase 4 activates apoptosis via the AKT/apoptosis signal-regulating kinase 1 pathway in pancreatic cancer PANC-1 cells. *Oncogene*.

[13] El-Kalla M, Onyskiw C, Baksh S (2010). Functional importance of RASSF1A microtubule localization and polymorphisms. *Oncogene*.

[14] Avruch J, Xavier R, Bardeesy N (2009). Rassf family of tumor suppressor polypeptides. *Journal of Biological Chemistry*.

[15] Pelosi G, Fumagalli C, Trubia M (2010). Dual role of RASSF1 as a tumor suppressor and an oncogene in neuroendocrine tumors of the lung. *Anticancer Research*.

[16] Kitagawa D, Kajiho H, Negishi T (2006). Release of *RASSF1C* from the nucleus by Daxx degradation links DNA damage and SAPK/JNK activation. *The EMBO Journal*.

[17] Vos MD, Martinez A, Elam C (2004). A role for the RASSF1A tumor suppressor in the regulation of tubulin polymerization and genomic stability. *Cancer Research*.

[18] Rong R, Jin W, Zhang J, Sheikh MS, Huang Y (2004). Tumor suppressor RASSF1A is a microtubule-binding protein that stabilizes microtubules and induces G_2_/M arrest. *Oncogene*.

[19] Van Der Weyden L, Tachibana KK, Gonzalez MA (2005). The RASSF1A isoform of RASSF1 promotes microtubule stability and suppresses tumorigenesis. *Molecular and Cellular Biology*.

[20] Foley CJ, Freedman H, Choo SL (2008). Dynamics of RASSF1A/MOAP-1 association with death receptors. *Molecular and Cellular Biology*.

[21] Harjes E, Harjes S, Wohlgemuth S (2006). GTP-Ras disrupts the intramolecular complex of C1 and RA domains of Nore1. *Structure*.

[22] Kashuba VI, Pavlova TV, Grigorieva EV (2009). High mutability of the tumor suppressor genes RASSF1 and RBSP3 (CTDSPL) in cancer. *PLoS One*.

[23] Schagdarsurengin U, Seidel C, Ulbrich EJ, Kölbl H, Dittmer J, Dammann R (2005). A polymorphism at codon 133 of the tumor suppressor RASSF1A is associated with tumorous alteration of the breast. *International Journal of Oncology*.

[24] Dammann R, Li C, Yoon JH, Chin PL, Bates S, Pfeifer GP (2000). Epigenetic inactivation of a RAS association domain family protein from the lung tumour suppressor locus 3p21.3. *Nature Genetics*.

[25] Agathanggelou A, Honorio S, Macartney DP (2001). Methylation associated inactivation of RASSF1A from region 3p21.3 in lung, breast and ovarian tumours. *Oncogene*.

[26] Burbee DG, Forgacs E, Zochbauer-Muller S (2001). Epigenetic inactivation of RASSF1A in lung and breast cancers and malignant phenotype suppression. *Journal of the National Cancer Institute*.

[27] Dallol A, Agathanggelou A, Fenton SL (2004). RASSF1A interacts with microtubule-associated proteins and modulates microtubule dynamics. *Cancer Research*.

[28] Bergqvist J, Latif A, Roberts SA (2010). RASSF1A polymorphism in familial breast cancer. *Familial Cancer*.

[29] Gao B, Xie XJ, Huang C (2008). RASSF1A polymorphism A133S is associated with early onset breast cancer in BRCA1/2 mutation carriers. *Cancer Research*.

[30] Lusher ME, Lindsey JC, Latif F (2002). Biallelic epigenetic inactivation of the RASSF1A tumor suppressor gene in medulloblastoma development. *Cancer Research*.

[31] Pan ZG, Kashuba VI, Liu XQ (2005). High frequency somatic mutations in RASSF1A in nasopharyngeal carcinoma. *Cancer biology & therapy*.

[32] Kanzaki H, Hanafusa H, Yamamoto H (2006). Single nucleotide polymorphism at codon 133 of the RASSF1 gene is preferentially associated with human lung adenocarcinoma risk. *Cancer Letters*.

[33] Min SS, Jin SC, Su JS, Yang TH, Lee H, Lim DS (2005). The centrosomal protein RAS association domain family protein 1A (RASSF1A)-binding protein 1 regulates mitotic progression by recruiting RASSF1A to spindle poles. *Journal of Biological Chemistry*.

[34] Liu L, Tommasi S, Lee DH, Dammann R, Pfeifer GP (2003). Control of microtubule stability by the RASSF1A tumor suppressor. *Oncogene*.

[35] Moshnikova A, Frye J, Shay JW, Minna JD, Khokhlatchev AV (2006). The growth and tumor suppressor NORE1A is a cytoskeletal protein that suppresses growth by inhibition of the ERK pathway. *Journal of Biological Chemistry*.

[36] Cuschieri L, Nguyen T, Vogel J (2007). Control at the cell center: the role of spindle poles in cytoskeletal organization and cell cycle regulation. *Cell Cycle*.

[37] Raynaud-Messina B, Merdes A (2007). Gamma-tubulin complexes and microtubule organization. *Current Opinion in Cell Biology*.

[38] Tommasi S, Dammann R, Zhang Z (2005). Tumor susceptibility of RASSF1A knockout mice. *Cancer Research*.

[39] Liu L, Vo A, McKeehan WL (2005). Specificity of the methylation-suppressed A isoform of candidate tumor suppressor RASSF1 for microtubule hyperstabilization is determined by cell death inducer C19ORF5. *Cancer Research*.

[40] Man C, Rosa J, Lee LTO (2007). Latent membrane protein 1 suppresses RASSF1A expression, disrupts microtubule structures and induces chromosomal aberrations in human epithelial cells. *Oncogene*.

[41] Mondello C, Scovassi AI (2010). Apoptosis: a way to maintain healthy individuals. *Sub-Cellular Biochemistry*.

[42] Van Herreweghe F, Festjens N, Declercq W, Vandenabeele P (2010). Tumor necrosis factor-mediated cell death: to break or to burst, that’s the question. *Cellular and Molecular Life Sciences*.

[43] Pennarun B, Meijer A, de Vries EGE, Kleibeuker JH, Kruyt F, de Jong S (2010). Playing the DISC: turning on TRAIL death receptor-mediated apoptosis in cancer. *Biochimica et Biophysica Acta*.

[44] Thorburn A (2004). Death receptor-induced cell killing. *Cellular Signalling*.

[45] Yu J, Ni M, Xu J (2002). Methylation profiling of twenty promoter-CpG islands of genes which may contribute to hepatocellular carcinogenesis. *BMC Cancer*.

[46] Schagdarsurengin U, Wilkens L, Steinemann D (2003). Frequent epigenetic inactivation of the RASSF1A gene in hepatocellular carcinoma. *Oncogene*.

[47] Jing F, Yuping W, Yong C (2010). CpG island methylator phenotype of multigene in serum of sporadic breast carcinoma. *Tumor Biology*.

[48] Fischer JR, Ohnmacht U, Rieger N (2006). Promoter methylation of RASSF1A, RAR beta and DAPK predict poor prognosis of patients with malignant mesothelioma. *Lung Cancer*.

[49] Kang TB, Ben-Moshe T, Varfolomeev EE (2004). Caspase-8 serves both apoptotic and nonapoptotic roles. *Journal of Immunology*.

[50] Bialik S, Kimchi A (2006). The death-associated protein kinases: structure, function, and beyond. *Annual Review of Biochemistry*.

[51] Gozuacik D, Kimchi A (2006). DAPk protein family and cancer. *Autophagy*.

[52] Mor I, Carlessi R, Ast T, Feinstein E, Kimchi A (2011). Death-associated protein kinase increases glycolytic rate through binding and activation of pyruvate kinase. *Oncogene*.

[53] Bialik S, Berissi H, Kimchi A (2008). A high throughput proteomics screen identifies novel substrates of death-associated protein kinase. *Molecular and Cellular Proteomics*.

[54] Verma SK, Ganesan TS, Parker PJ (2008). The tumour suppressor RASSF1A is a novel substrate of PKC. *FEBS Letters*.

[55] Song SJ, Kim SJ, Song MS, Lim DS (2009). Aurora B-mediated phosphorylation of RASSF1A maintains proper cytokinesis by recruiting syntaxin16 to the midzone and midbody. *Cancer Research*.

[56] Demon D, Van Damme P, Berghe TV (2009). Caspase substrates: easily caught in deep waters?. *Trends in Biotechnology*.

[57] Riedl SJ, Shi Y (2004). Molecular mechanisms of caspase regulation during apoptosis. *Nature Reviews Molecular Cell Biology*.

[58] Baksh S, Tommasi S, Fenton S (2005). The tumor suppressor RASSF1A and MAP-1 link death receptor signaling to bax conformational change and cell death. *Molecular Cell*.

[59] Ghazaleh HA, Chow RS, Choo SL (2010). 14-3-3 mediated regulation of the tumor suppressor protein, RASSF1A. *Apoptosis*.

[60] Vos MD, Dallol A, Eckfeld K (2006). The RASSF1A tumor suppressor activates bax via MOAP-1. *Journal of Biological Chemistry*.

[61] Liu L, Xie R, Yang C, McKeehan WL (2009). Dual function microtubule- and mitochondria-associated proteins mediate mitotic cell death. *Cellular Oncology*.

[62] Donninger H, Allen N, Henson A (2011). Salvador protein is a tumor suppressor effector of RASSF1A with hippo pathway-independent functions. *Journal of Biological Chemistry*.

[63] Yi M, Yang J, Chen X (2011). RASSF1A suppresses melanoma development by modulating apoptosis and cell-cycle progression. *Journal of Cellular Physiology*.

[64] Song MS, Song SJ, Ayad NG (2004). The tumour suppressor RASSF1A regulates mitosis by inhibiting the APC-Cdc20 complex. *Nature Cell Biology*.

[65] Whitehurst AW, Ram R, Shivakumar L, Gao B, Minna JD, White MA (2008). The RASSF1A tumor suppressor restrains anaphase-promoting complex/cyclosome activity during the G_1_/S phase transition to promote cell cycle progression in human epithelial cells. *Molecular and Cellular Biology*.

[66] Liu L, Baier K, Dammann R, Pfeifer GP (2007). The tumor suppressor RASSF1A does not interact with Cdc20, an activator of the anaphase-promoting complex. *Cell Cycle*.

[67] Chow C, Wong N, Pagano M (2011). Regulation of APC/C(Cdc20) activity by RASSF1A-APC/C(Cdc20) circuitry. *Oncogene*.

[68] Shivakumar L, Minna J, Sakamaki T, Pestell R, White MA (2002). The RASSF1A tumor suppressor blocks cell cycle progression and inhibits cyclin D1 accumulation. *Molecular and Cellular Biology*.

[69] Fenton SL, Dallol A, Agathanggelou A (2004). dentification of the E1A-regulated transcription factor p120 E4F as an interacting partner of the RASSF1A candidate tumor suppressor gene. *Cancer Research*.

[70] Jiang L, Rong R, Sheikh MS, Huang Y (2011). Cullin-4A.DNA damage-binding protein 1 E3 ligase complex targets tumor suppressor RASSF1A for degradation during mitosis. *Journal of Biological Chemistry*.

[71] Hamilton G, Yee KS, Scrace S, O’Neill E (2009). ATM regulates a RASSF1A-dependent DNA damage response. *Current Biology*.

[72] Dreijerink K, Braga E, Kuzmin I (2001). The candidate tumor suppressor gene, RASSF1A, from human chromosome 3p21.3 is involved in kidney tumorigenesis. *Proceedings of the National Academy of Sciences of the United States of America*.

[73] Kim ST, Lim DS, Canman CE, Kastan MB (1999). Substrate specificities and identification of putative substrates of ATM kinase family members. *Journal of Biological Chemistry*.

[74] Zhou X, Li TT, Feng X (2012). Targeted polyubiquitylation of RASSF1C by the Mule and SCF beta-TrCP ligases in response to DNA damage. *Biochemical Journal*.

[75] Li J, Wang F, Protopopov A (2004). Inactivation of RASSF1C during *in vivo* tumor growth identifies it as a tumor suppressor gene. *Oncogene*.

[76] Amaar YG, Minera MG, Hatran LK, Strong DD, Mohan S, Reeves ME (2006). Ras association domain family 1C protein stimulates human lung cancer cell proliferation. *American Journal of Physiology*.

[77] Reeves ME, Baldwin SW, Baldwin ML (2010). Ras-association domain family 1C protein promotes breast cancer cell migration and attenuates apoptosis. *BMC Cancer*.

[78] Estrabaud E, Lassot I, Blot G (2007). RASSF1C, an isoform of the tumor suppressor RASSF1A, promotes the accumulation of {beta}-catenin by interacting with {beta}TrCP. *Cancer Research*.

[79] Malpeli G, Amato E, Dandrea M (2011). Methylation-associated down-regulation of ,RASSF1A and up-regulation of RASSF1C in pancreatic endocrine tumors. *BMC Cancer*.

[80] Knudson AG (1971). Mutation and cancer: statistical study of retinoblastoma. *Proceedings of the National Academy of Sciences of the United States of America*.

[81] Dammann R, Schagdarsurengin U, Seidel C (2005). The tumor suppressor RASSF1A in human carcinogenesis: an update. *Histology and Histopathology*.

